# Novel technologies and emerging biomarkers for personalized cancer immunotherapy

**DOI:** 10.1186/s40425-016-0107-3

**Published:** 2016-01-19

**Authors:** Jianda Yuan, Priti S. Hegde, Raphael Clynes, Periklis G. Foukas, Alexandre Harari, Thomas O. Kleen, Pia Kvistborg, Cristina Maccalli, Holden T. Maecker, David B. Page, Harlan Robins, Wenru Song, Edward C. Stack, Ena Wang, Theresa L. Whiteside, Yingdong Zhao, Heinz Zwierzina, Lisa H. Butterfield, Bernard A. Fox

**Affiliations:** Memorial Sloan-Kettering Cancer Center, 1275 New York Ave Box 386, New York, NY 10065 USA; Genentech, Inc., 1 DNA Way South, San Francisco, CA 94080 USA; Bristol-Myers Squibb, 3551 Lawrenceville Road, Princeton, NJ 08648 USA; Center of Experimental Therapeutics and Ludwig Institute of Cancer Research, University Hospital of Lausanne, Rue du Bugnon 21, 1011 Lausanne, Switzerland; Department of Pathology, University of Athens Medical School, “Attikon” University Hospital, 1st Rimini St, 12462 Haidari, Greece; Epiontis GmbH, Rudower Chaussee 29, 12489 Berlin, Germany; Netherlands Cancer Institute, Postbus 90203, 1006 BE Amsterdam, Netherlands; Italian Network for Biotherapy of Tumors (NIBIT)-Laboratory, c/o Medical Oncology and Immunotherapy, University Hospital of Siena, V.le Bracci,16, Siena, 53100 Italy; Stanford University Medical Center, 299 Campus Drive, Stanford, CA 94303 USA; Earle A. Chiles Research Institute, Providence Cancer Center, 4805 NE Glisan Street, Portland, OR 97213 USA; Adaptive Technologies, Inc., 1551 Eastlake Avenue East Suite 200, Seattle, WA 98102 USA; AstraZeneca, One MedImmune Way, Gaithersburg, MD 20878 USA; PerkinElmer, 68 Elm Street, Hopkinton, MA 01784 USA; Sidra Medical and Research Center, PO Box 26999, Doha, Qatar; University of Pittsburgh Cancer Institute, 5117 Centre Ave, Suite 1.27, Pittsburgh, PA 15213 USA; National Cancer Institute, 9609 Medical Center Drive, Rockville, MD 20850 USA; Innsbruck Medical University, Medizinische Klinik, Anichstrasse 35, Innsbruck, A-6020 Austria; Department of Medicine, Surgery and Immunology, University of Pittsburgh Cancer Institute, 5117 Centre Avenue, Pittsburgh, PA 15213 USA

**Keywords:** Immune checkpoint blockade, Cancer immunotherapy, Biomarkers, Task Force, Immune monitoring, Technology, Bioinformatics

## Abstract

The culmination of over a century’s work to understand the role of the immune system in tumor control has led to the recent advances in cancer immunotherapies that have resulted in durable clinical responses in patients with a variety of malignancies. Cancer immunotherapies are rapidly changing traditional treatment paradigms and expanding the therapeutic landscape for cancer patients. However, despite the current success of these therapies, not all patients respond to immunotherapy and even those that do often experience toxicities. Thus, there is a growing need to identify predictive and prognostic biomarkers that enhance our understanding of the mechanisms underlying the complex interactions between the immune system and cancer. Therefore, the Society for Immunotherapy of Cancer (SITC) reconvened an Immune Biomarkers Task Force to review state of the art technologies, identify current hurdlers, and make recommendations for the field. As a product of this task force, Working Group 2 (WG2), consisting of international experts from academia and industry, assembled to identify and discuss promising technologies for biomarker discovery and validation. Thus, this WG2 consensus paper will focus on the current status of emerging biomarkers for immune checkpoint blockade therapy and discuss novel technologies as well as high dimensional data analysis platforms that will be pivotal for future biomarker research. In addition, this paper will include a brief overview of the current challenges with recommendations for future biomarker discovery.

## Background

The role of the immune system in cancer control has been debated for over a century. The field of cancer immunology has progressed with knowledge obtained from animal studies, accumulated clinical observations and translational research. Interestingly, starting in the 1900s and every 50 years thereafter, three main theories had been proposed to refine our understanding of the impact of the immune system on cancer. The first theory was suggested by Paul Ehrlich’s human protective cancer immunity, followed by Burnet and Thomas’s concept of “cancer immunosurveillance”, and recently by Schreiber, Old and Smyth’s “cancer immunoediting” [1–4]. “Cancer immunosurveillance” originally implied that the immune system was involved at the initial stages of cellular transformation and played a solely protective role. Now, the term “cancer immunoediting” is used to better describe the protective activities, positive and negative sculpting actions of the immune response on developing tumors in a continuous manner. This process can potentially result in the complete elimination of some tumors, but it can also generate a non-protective immune state to others that may favor the development of immunologic evasion. Meanwhile, our perception of cancer has changed dramatically. In the past, tumors were thought to be a result of a single, clonal, disordered cell, when in actuality, most resulted from multiple (pre)malignant cells [5]. Tumors are comprised of heterogeneous cell populations, including transformed cells and untransformed cells (such as stromal, endothelial and immune cells), which have indispensable functions in their microenvironment [6, 7]. The evasion of immune destruction is now commonly accepted as a hallmark of cancer [8]. Therefore, understanding the status and interaction between cancer and the immune system in the tumor microenvironment (TME) is of importance for cancer immunotherapy strategies.

T cell infiltration in certain human tumors is associated with an improved clinical outcome [9, 10]. The accumulating evidence suggests that tumors can be classified into two groups: Immunologically-ignorant tumors and immunologically-responsive tumors (or non-inflamed tumors vs T cell-inflamed tumors), based on the presence or absence of immune cell infiltrations [11]. Clinically detectable tumors have usually already evolved mechanisms to evade an immune response. The tumor immune escape mechanism may be different for each type, which may involve one or multiple steps of the cancer-immunity cycle [12]. Immunologically-ignorant tumors may be caused by a low mutation load, immune tolerance against self-antigens and lack of essential chemokines and other molecules for T cell homing into tumor sites. In contrast, the progression of immunologically-responsive tumors with T cell infiltration indicates an insufficient response that is probably due to intrinsic T cell immune-inhibition and extrinsic tumor-related T cell immunosuppression [13]. Intrinsic T cell immunosuppression involves anergy and exhaustion of activated T cells with endogenous immune checkpoint molecules including cytotoxic T lymphocyte-associated antigen 4 (CTLA-4), programmed cell death 1 (PD-1), T cell immunoglobulin mucin-3 (Tim-3) and lymphocyte-activation gene 3 (LAG-3) [14]. The secretion of extrinsic inhibitory molecules such as TGF-β, IL-10 and indoleamine 2,3-dioxyenase (IDO) could have a direct negative impact on T cell function in the TME and on the recruitment of anti-inflammatory cells, including tolerogenic antigen presenting cells, regulatory T cells (Treg) and myeloid derived suppressor cells (MDSC). These suppressive immune cells can also inhibit the actions of cytotoxic T lymphocytes [13].

It is a promising approach to block these immunosuppressive mechanisms to augment the function of endogenous antitumor T cells, which can deliver a robust and effective clinical response. Blockade antibodies against T cell checkpoint molecules including CTLA-4 and the PD-1/PD-L1 axis in mono- or combination therapies have begun to revolutionize the current standard cancer treatment in various cancer types, such as melanoma, non-small cell lung cancer (NSCLC), bladder cancer and Hodgkin’s lymphoma [15–22]. Immunologically-responsive tumors are more likely to respond to these checkpoint blockade antibody therapies than immunologically-ignorant tumors [11]. Moreover, prior interventions to achieve local, productive inflammation in the TME are required in combination with these therapies to enhance the clinical response for immunologically-ignorant tumors. Thus, it is essential to perform biomarker studies to further characterize these different classes of tumors and provide guidance for therapeutic strategies.

**Fig. 1:**
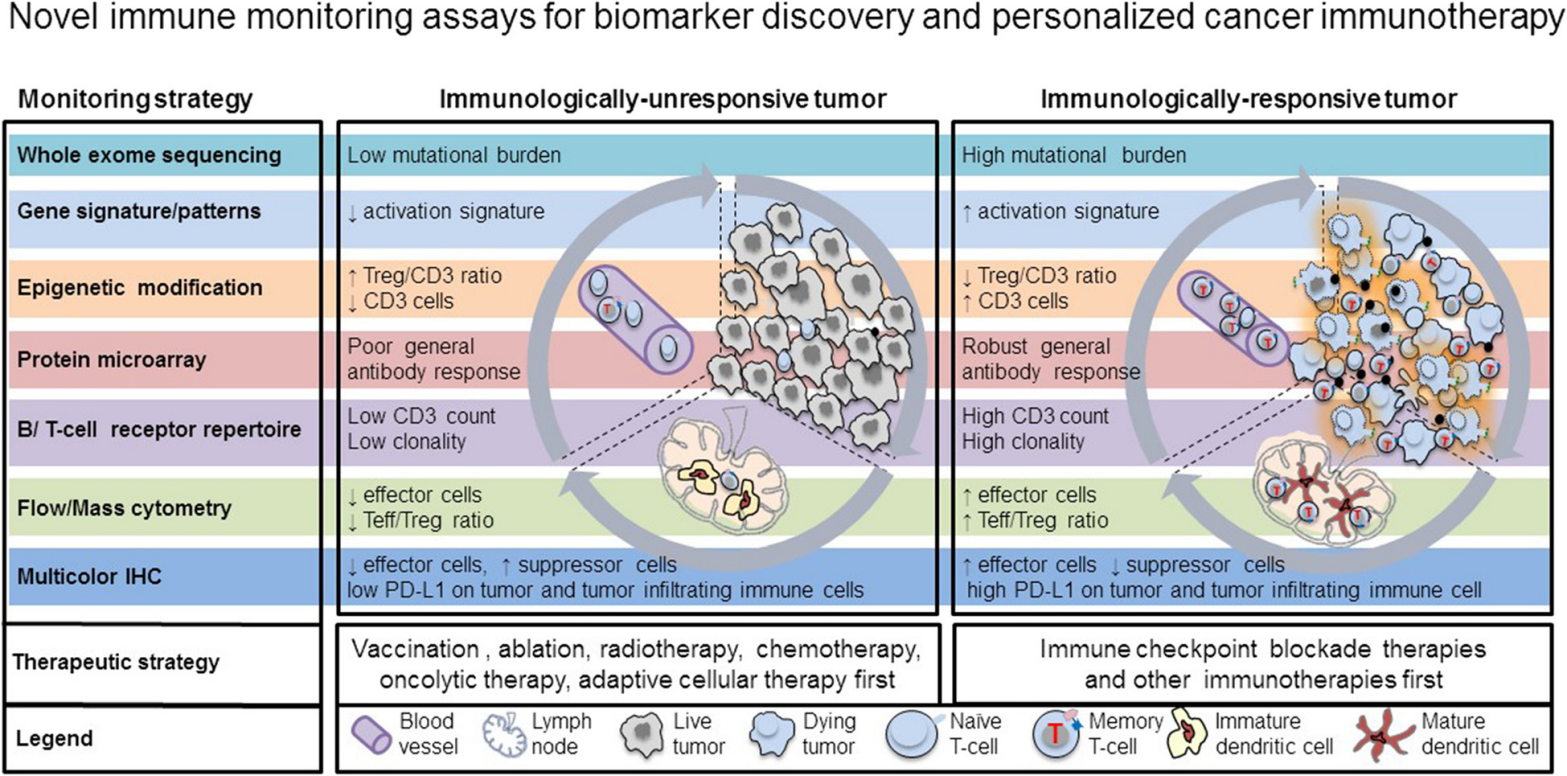
High-throughput immune assessment for biomarker discovery and personalized cancer immunotherapy. Immunologically-ignorant and immunologically-responsive tumors are classified by the presence of immune cells in the tumor microenvironment. Potential biomarkers identified from high-throughput technologies can further differentiate these tumors by the mutation load, gene/protein/antibody signature profile, phenotype and function of immune cells, and can also provide clinical strategies for personalized cancer immunotherapies. The new and innovative technologies that can be utilized to identify potential biomarkers include whole exome sequencing, gene signature, epigenetic modification, protein microarray, B/T cell receptor repertoire, flow/mass cytometry and multicolor IHC. *Arrows* indicate a decrease (↓) or increase (↑)

Improved high-throughput technologies are providing feasible tools for analyzing the mutation antigen profile, the gene signature and epigenetic modification of tumor and immune cells, the breadth of antibody responses, as well as the magnitude, homing capacity, cytotoxic function and T cell receptor (TCR) repertoire of T lymphocytes. These novel technologies will help advance precision medicine [23]. New technological approaches will enable us to identify predictive biomarkers such as immunologic signatures or profiles for the patients who will most likely benefit from current immunotherapies. In addition, they will help patients avoid immune-related adverse events or adverse events of special interest and reduce treatment costs for those unlikely to respond [17]. Furthermore, they will enhance our understanding of the mechanisms underlying cancer immunotherapies and aid in the development of more appropriate therapies for specific patient populations. In this paper, we will discuss the current progress to identify biomarkers for immune checkpoint blockade therapies as well as novel technologies and their potential application for future cancer immunotherapy biomarker discoveries, as illustrated in Fig. 1.

## Review

### Emerging biomarkers for CTLA-4 immune checkpoint blockade immunotherapy

Immune checkpoint blockade has led to durable antitumor effects in patients with metastatic melanoma, NSCLC and other tumor types [15, 17, 24–29]. Ipilimumab, an antibody that blocks CTLA-4, was approved by the U.S. Food and Drug Administration (FDA) for patients with advanced melanoma in 2011. However, although a subset of patients benefit, it is often with delayed radiographic response and at the expense of mechanism-based toxicity [17]. Therefore, it is imperative to identify biomarkers in order to elucidate the pharmacodynamic changes, understand the potential mechanisms of action and to find new correlates associated with clinical benefits and/or toxicities.

Several serum markers such as lactate dehydrogenase (LDH), C-reactive protein, vascular endothelial growth factor (VEGF) and soluble CD25 are associated with clinical outcome in advanced melanoma patients treated with ipilimumab [30–34]. In addition, a variety of assays are available to monitor phenotypic changes in immune cells such as human leukocyte antigen (HLA)-DR and activated inducible co-stimulator (ICOS) on T cells, to measure changes in target immune cell populations such as MDSC and to assess tumor associated antigen (TAA) specific responses as well as evaluate the functionality and gene expression profile of antigen-specific T cell populations. These assays have led to preliminary findings of potential emerging biomarkers for CTLA-4 blockade therapy as described in the following section.

Ipilimumab augments antitumor immune responses by activating and increasing the proliferation of T cells [35]. Thus, absolute lymphocyte count (ALC) is a potential pharmacodynamic biomarker for ipilimumab treatment in patients with melanoma and other solid tumors [36–38]. Following treatment with ipilimumab, an ALC ≥1000/μL at week 7 or an increase in ALC between baseline and week 12 was significantly associated with longer overall survival [33, 39, 40]. Because the ALC contains a variable heterogeneous lymphocyte population as a general biomarker, there has been strong interest in characterizing changes in specific T cell subsets during CTLA-4 blockade therapy. Increased levels of HLA-DR, CD45RO, central memory markers (CCR7^+^CD45RA^−^) and effector memory markers (CCR7^−^CD45RA^−^) on CD4^+^ and CD8^+^ T cells were reported after ipilimumab treatment in several studies [41–45]. However, the elevation of these T cell markers did not correlate with clinical response to ipilimumab.

ICOS is expressed on the cell surface of activated T cells and plays a role in T cell expansion and survival. The frequency of CD4^+^ICOS^+^ T cells was shown to increase in a dose-dependent manner in patients with bladder cancer, breast cancer and mesothelioma after treatment with either ipilimumab or tremelimumab [45–49]. In addition, a sustained increase in CD4^+^ICOS^+^ T cells was observed over 12 weeks after CTLA-4 blockade therapy and correlated with improved survival in four independent studies [46, 49–51]. Therefore, an increase in the frequency of CD4^+^ICOS^+^ T cell may be a reproducible pharmacodynamic biomarker to indicate biological activity for CTLA-4 blockade therapy [52]. However, it would be worthwhile to prospectively investigate changes in the frequency of multiple T cell subsets in relation to CTLA-4 blockade therapy in a large cohort of patients.

Cancers are immunogenic and express a variety of TAAs. CTLA-4 blockade was shown to potentiate the production of TAA-specific antibodies as well as a CD4^+^ and CD8^+^ antigen-specific T cell response in patients with melanoma, ovarian and prostate cancer [45, 53–56]. Moreover, melanoma patients seropositive for the cancer-testis antigen NY-ESO-1 were more likely to experience clinical benefit than those who were seronegative [57]. In contrast, there was no significant association between humoral response to tumor antigens and clinical benefit in two other studies [45, 58]. However, because of small sample size, different response criteria and varying doses of ipilimumab, it is ultimately difficult to make any specific conclusions based on these studies alone. CTLA-4 blockade has also been shown to actually potentiate a robust spectrum of tumor specific antibody responses. For advanced prostate cancer, it was shown that patients who clinically responded to CTLA-4 blockade also developed an enhanced antibody response to a greater number of endogenous antigens than non-responders. In this study, the majority of antibody responses were patient specific, although there were some shared antibody responses among clinical responders [56]. Further prospective validation is warranted to characterize the tumor antigen specific antibody response as potential biomarker for anti-CTLA-4 therapy.

The evaluation of TAA-specific T cell response has also been an intense focus of immune monitoring for anti-CTLA-4 immunotherapies. A high frequency of Melan-A or NY-ESO-1 specific CD8^+^ T cells were detected in melanoma and prostate cancer patients who showed a clinical response to anti-CTLA-4 therapy [55, 59]. In these patients, the presence of integrated antibody and the CD8+ T cell response to NY-ESO-1 was associated with a significant survival advantage [57, 60]. In addition, a recent study also reported that ipilimumab induced a significant increase in the number of newly detected melanoma-reactive T cells by enhancing T cell priming [61]. Moreover, tumor genetics were shown to be important in defining clinical benefit in ipilimumab treated melanoma patients [62, 63]. The expression of immune-related genes in pretreatment tumor biopsy specimens, especially interferon gamma responsive genes, was correlated positively with clinical activity in ipilimumab-treated melanoma patients [64]. A recent study showed that two cytolytic genes (granzyme A and perforin) in the TME were significantly enriched in the ipilimumab clinical benefit cohort compared to the cohort that showed no clinical benefit [63]. These new findings suggest that the antitumor effect of CTLA-4 blockade likely involves the amplification of a preexisting or the priming and induction of an immune response against various antigenic targets, especially mutant genes.

MDSC are a phenotypically heterogeneous cell population that is comprised of myeloid-cell progenitors and precursors of myeloid cells. Interestingly, MDSC can also function as antigen-presenting cells (APCs). Human MDSC have been identified in patients with pancreatic cancer, breast cancer, NSCLC and head and neck squamous cell carcinoma [65, 66]. Human MDSC have an immature phenotype that is typically lineage negative (Lin^−^), CD14^−^, HLA-DR^−^, CD15^+^, CD34^+^, CD11b^+^, CD33^+^ and CD13^+^ [67, 68]. MDSC exert an immunosuppressive function mainly through the production of suppressive molecules, such as ARG1, cytokines, transforming growth factor-beta (TGF- β) or IL-10. The common proposed phenotype in humans is CD14^+^/HLA-DR^low/-^, which is based upon this cell population’s ability to suppress lymphocyte function. The number of CD14^+^/HLA-DR^low/-^ cells was shown to be elevated in melanoma patients and this increase correlated with melanoma disease activity [69]. Therefore, MDSC have been recently proposed as a potential biomarker associated with disease progression or survival [70]. Ipilimumab treatment induced an early decrease in the frequency of MDSC [71]. In addition, a lower baseline MDSC frequency was associated with improved overall survival [72, 73].

Overall, the biomarkers for CTLA-4 blockade therapy were mostly identified from small cohort studies. Thus, ongoing efforts are needed to validate these findings in a larger cohort of patients in prospective clinical trials and to determine whether these findings are specific to ipilimumab treatment compared with other cancer immunotherapies. Ultimately, the development of robust and validated biomarkers that are predictive and/or prognostic will help guide future clinical trials. Novel high-throughput technologies, such as exome sequencing, flow-based phenotyping and multifunctional assays and T cell receptor analysis, have advanced recent antigen specific biomarker discovery and will provide more tools in order to validate the emerging biomarkers for CTLA-4 blockade therapy. These new technologies and their potential application will be discussed in details in subsequent sections.

### Regulatory T cells as potential biomarkers

The accumulation of Treg and MDSC in human tumors and their increased frequency in the peripheral circulation of cancer patients have been widely reported [74, 75]. Many reports, but not all, link these accumulations of CD4^+^FOXP3^+^CD25^hi^ Treg to poor prognosis due to the suppression of antitumor immune response by the Treg [74]. However, in human solid tumors such as colorectal cancer or breast carcinomas, which are often richly infiltrated with immune cells, the presence and density of FOXP3^+^ Treg have been reported to predict favorable outcome and a better local regional control of the tumor [76]. Given recent emphasis on the tumor “immune signature” and emerging correlations of the immunohistology data to cancer patients’ survival [9, 76], the reliable phenotypic, and especially functional, characterization of Treg in situ and in body fluids is of critical importance. To date, most of the studies examining the association between Treg phenotype and prognosis or therapeutic response are still based on the use of FOXP3 as a “specific” Treg marker [77]. Recent data show that FOXP3 is not a reliable marker of human Treg and that a collection of several other markers may be a better option [77, 78].

In general, human Treg have been difficult to study for the following reasons: (1) they represent only a minor subset of CD4^+^ T cells (about 5 %) and thus are often limited in numbers; (2) they lack a specific surface marker, making their isolation and identification questionable; and (3) Treg plasticity has made it difficult to differentiate naïve (nTreg) or thymus-derived Treg (tTreg) from inducible (iTreg) or peripheral Treg (pTreg). Various marker panels used to phenotypically identify Treg invariably include expression of CD25^hi^ and/or FOXP3^+^. In addition, the absence of CD127 or CD26 has been useful for Treg typing and isolation [79, 80].

The following recommendations for Treg flow cytometry panels have recently been made: (1) a minimal definition of Treg should include CD3, CD4, CD25, CD127, FOXP3 markers with Ki67 and CD45RA to clarify the Treg activation status; (2) the sole dependence on any of the three most commonly used flow panels for the Treg phenotypic definition [(a) CD25^+^CD127^low^ FOXP3^+^Treg; (b) FOXP3^+^HELIOS^+^Treg; or (c) FOXP3^hi^CD45RA^neg^ vs. FOXP3^int^CD45RA^+^ to distinguish activated from naïve Treg, respectively] leads to an underestimation of the Treg frequency ranging from 25 to 65 %. Functional markers, such as CD39 and CTLA-4, denote activated or iTreg, and thus, may be considered “optional” markers.

It has been reported that expression of surface markers on Treg becomes altered in disease [81] and in patients undergoing conventional therapies or immune therapies [82, 83]. Therefore, the selection of a panel of markers for measuring Treg is a critical task that will ultimately determine its role as a prognostic biomarker in cancer and other disease. As in cancer, iTreg are undoubtedly the predominant Treg subset in situ and in the peripheral circulation; their number, localization and functions are of utmost importance. Thus, “activation” markers, such as CD39, CTLA-4, latency-associated peptide (LAP), glycoprotein A repetitions predominant (GARP), PD-1, PD-L1 and others that are often overexpressed on Treg in cancer, emerge as important surrogate markers for Treg function and should be included in the monitoring of Treg in cancer patients. Although these markers are not specific to Treg, they are useful when used in combination with CD25^hi^ and FOXP3^+^ to assess the functional potential of Treg by flow cytometry and eliminate the need for Treg isolation that is necessary in conventional carboxyfluoresceinsuccinimidyl aster (CFSE)-based suppressor assays [84, 85]. Efforts to identify a specific Treg marker that might distinguish nTreg from iTreg have recently focused on Kruppel-like factor 2 (KLF2), a transcription factor that regulates chronic inflammation and is necessary for the development of iTreg but not of nTreg [86]. Although there is still no consensus on which marker panel (of the several listed above) is best and which subset of Treg should be monitored, some investigators prefer to focus on one functional subset, e.g., the CD4^+^CD39^+^CD25^+^ adenosine-producing Treg [87]. Based on the principle that function rather than phenotype determines the biological and clinical significance of Treg, this strategy, while limited in scope, offers the advantage of following disease-associated changes in a single subset of Treg and correlating these changes to disease progression [87].

A number of in vitro suppression assays are available for human Treg [78]. Among these, flow cytometry based assays to measure the surface expression of LAP/GARP on Treg, the intracellular expression of inhibitory cytokines (TGF-β or IL-10) or the downregulation of CD69 or CD154 expression in co-incubated responder cells require a short-term, ex vivo activation of Treg. In this regard, the flow-based assays are easier and have a higher throughput potential than the conventional co-culture assays of Treg with CFSE-labeled responder cells.

Treg have constitutive expression of FOXP3 and CTLA-4 on their cell surface and intracellular. Recently, a study illustrated that anti-CTLA-4 antibody depleted Treg in tumor lesions through Fc-dependent mechanism to potentially enhance antitumor immunity in mice [88]. Moreover, the number of Treg (CD4^+^CD25^+^CD62L^+^ cells) in peripheral blood decreased at early time points but rebounded to a level at or above baseline value at the time of next dose [89]. In contrast, several studies reported that ipilimumab in fact induced the proliferation and expansion of Treg, especially at lower doses, whereas activated effector CD4^+^ cells were expanded only at higher ipilimumab doses [43, 90]. Although the decreasing FOXP3/Treg was associated with a better clinical outcome in ipilimumab treated melanoma patients [91], it will be critical to further characterize Treg with new markers as described above in both peripheral blood and tumor tissue and explore their correlation with clinical outcome in patients treated with immunotherapies.

### Emerging biomarkers for PD-1/PD-L1 immune checkpoint blockade immunotherapy

The immune checkpoint molecule programmed death-1 (PD-1, CD279) is upregulated on activated T lymphocytes and inhibits T-cell function by binding to its ligands PD-L1 (B7-H1, CD274) and PD-L2 (B7-DC, CD272) [92–94]. The PD-1/PD-L1 axis plays a pivotal role in regulating T cell activation and peripheral immune tolerance. The PD-1/PD-L1 interaction functions to prevent bystander tissue damage during inflammation, but it can also maintain an immunosuppressive TME that allows tumor cells to evade immune surveillance [95, 96]. Similar to CTLA-4 blockade therapy, monoclonal antibodies that block PD-1 on immune effector cells or PD-L1 on tumor cells and/or APCs have been employed to restore immune activation. Several antibodies against PD-1 (nivolumab, pembrolizumab, MED10680, pidilizumab) and PD-L1 (BMS-936559, MED14736, atezolizumab, MSB0010718A) are currently under clinical investigation and have demonstrated generally manageable safety profiles and remarkable anti-tumor responses in cancer patients with a wide range of metastatic diseases [17, 18, 97–102]. As a result of positive clinical results in early studies, the FDA approved pembrolizumab and nivolumab for patients with advanced melanoma in 2014 and for patients with metastatic squamous and non-squamous NSCLC in 2015.

Although both are immune checkpoints, CTLA-4 and PD-1 have distinct roles in regulating immunity. CTLA-4 regulates the amplitude of early activation of naïve and memory T cells, while PD-1 with the corresponding upregulation of its ligands PD-L1 and PD-L2 limits the activity of T cells in the periphery during an inflammatory response [95, 96]. PD-1 plays multiple roles in immune regulation, and it is expressed on a variety of immune cell types, including T cells (CD8^+^ and CD4^+^), B cells, natural killer cells, monocytes and APCs. The PD-1/PD-L1 axis can negatively regulate the activation and function of T and B cells as well as inducing Treg, thereby inhibiting anti-tumor immunity. Moreover, PD-1 is expressed with other immune checkpoint molecules such as LAG-3 and Tim-3 on non-functional CD8^+^ T cells, which supports the notion that PD-1 may interact with other immune checkpoints to control anti-tumor immunity [99]. In addition to PD-1, PD-L1 also binds to B7.1 (CD80) and can inhibit T cell proliferation [100]. These distinct inhibitory interactions act more selectively within the TME, suggesting that anti-PD-1 and anti-PD-L1 antibodies may have different clinical activities and related toxicities compared with CTLA-4 blockade therapy. Research focused on identifying clinical biomarkers is necessary to elucidate the mechanisms underlying the PD-1/PD-L1 mediated blockade and to predict patient outcomes following anti-PD-1/PD-L1 therapies. Furthermore, it will also provide key insights to develop combinatorial therapeutic strategies for future clinical trials.

Studies to identify peripheral blood immune biomarkers have illustrated that PD-1/PD-L1 blockade increases effector T-cell proliferation (CD8^+^/HLA-DR^+^/Ki67^+^ T cells), the production of inducible T-cell alpha chemoattractant (ITAC), interferon-gamma (IFN-γ) and IL-18. However, in these studies there was no significant correlation between these markers and clinical response in patients [28, 29]. Because of the unique expression pattern and functional characterization of the PD-1/PD-L1 axis molecules, the local TME may be a key site for identifying predictive biomarkers for PD-1 pathway blockade. For example, the expression of PD-L1 in tumor-infiltrating immune cells analyzed by immunohistochemisty (IHC) is associated with response to atezolizumab in patients with bladder cancer [29]. Additionally, elevated expression of IFN-γ and IFN-γ-induced genes in pre-treatment tumors is associated with clinical response in patients with melanoma, although there was no such association reported in NSCLC or renal cell carcinoma [28]. Another study illustrated that melanoma patients who had a higher number of pre-treatment CD8^+^ T cells and TCR oligoclonality experienced a better clinical response to pembrolizumab [103]. Furthermore, it has been shown that immune PD-1/PD-L1 blockade has the capacity to enhance and sustain endogenous immunity against mutated tumor neoantigens, thereby achieving durable tumor control. Proof of principle studies in melanoma and NSCLC have shown that high mutational load is associated with clinical response in patients treated with anti-PD-1 antibodies [62, 104]. A recent study also illustrated that tumor mismatch repair status predicted the clinical benefit of immune checkpoint blockade with pembrolizumab [105]. Tumors with high mutational load are likely more immunogenic, which can persistently stimulate neoantigen-specific CD4^+^ and CD8^+^ T cells. Thus, these results suggest that tumor mutational load could be potential predictive biomarker for PD-1/PD-L1 blockade therapy.

PD-L1 is upregulated on many different tumor types to inhibit the local antitumor T cell response. Because it is functional only through the ligation with its counter-receptor, the cell surface, or membranous, expression of PD-L1 is essential for its biologic function. Two major potential mechanisms, known as adaptive and intrinsic resistance, can regulate the expression of PD-L1 on tumor cells [96, 102]. Adaptive resistance occurs when the tumor co-opts the natural physiology of the PD-1 pathway to enable its own protection. For example, the expression of PD-L1 is up-regulated on most epithelial cancers in response to various inflammatory mediators such as cytokine IFN-γ, IL-4, IL-10, LPS, GM-CSF and VEGF. Intrinsic resistance refers to constitutive genetic alternations or the activation of signaling pathways (e.g., PTEN loss, activation of MEK/ERK or MyD88 signaling or EGFR mutations) that drive the expression of PD-L1 on tumor cells [106–109]. Although it has been described, the prognostic significance of PD-L1 expression on tumor cells remains to be determined.

Both prospective and retrospective analyses in large Phase I, II and III trials in NSCLC and melanoma patients have demonstrated the association between tumor PD-L1 expression and response to anti-PD-1 agents [17, 98]. In addition, a correlation between PD-L1 expression in the TME and clinical responsive to PD-1 blockade has also been shown [28, 29, 110]. Interestingly, PD-L1 expression on tumor-infiltrating immune cells was significantly associated with clinical response in NSCLC patients treated with atezolizumab [28]. However, due to the complexities of PD-L1 IHC, further studies are needed to carefully validate these observation and other TME characteristics in either mono or combinatorial therapy settings [110]. In addition, due to the temporal changes in PD-L1 expression during the treatment, the immune profile and tumor signature need to be assessed at baseline. Moreover, measuring alterations in the TME during treatment will also be critical for future biomarker studies for PD-1/PD-L1 targeted immunotherapy. Despite these caveats, the current landscape for archival or pre-dose tumor PD-L1 as a predictive marker of efficacy for PD-L1/PD-1 targeted agents looks promising. Pembrolizumab was the first checkpoint inhibitor to get approved with a companion diagnostic test to measure tumor PD-L1 expression in 2^nd^ line + NSCLC. Patients with high tumor PD-L1 expression as defined by a proportional score of ≥50 % (PS ≥50 %) demonstrated improved objective response rates of 45 % compared with 19 % in all enrolled NSCLC patients [111]. Moreover, although it is not required for patient selection, nivolumab was approved for non-squamous NSCLC with the first complementary diagnostic test to measure tumor PD-L1 expression in order to help identify patients with a greater likelihood of improved survival [112]. However, because patients with PD-L1 negative tumors had comparable activity to docetaxel with a favorable tolerability profile and the overall intention-to-treat population in the trial was positive, nivolumab was approved for 2^nd^ line NSCLC, regardless of PD-L1 status [113]. PDL1 was not predictive of outcome for squamous NSCLC patients treated with nivolumab.

Currently, there are a number of emerging biomarkers for CTLA-4 and PD-1/PD-L1 immune checkpoint blockade therapies. However, progress to identify new and validate current biomarker candidates has been limited by the use of unstandardized assays that provide limited data and variable results. Recent technological advances in high-throughput techniques will not only allow potential biomarkers to be validated across large prospective studies, but will also facilitate the discovery of novel biomarkers and enhance our understanding of the mechanisms underlying cancer immunotherapies. Therefore, the remainder of this paper will focus on novel technologies and highlight the potential impact of each technology on current biomarker validation and future biomarker discovery.

### Whole exome sequencing for neoantigen discovery

Cancer is a genetic disease. The accumulation of genetic mutations in a tumor leads to a change in its proteome. A “cancer anti-genome” generated during this process can be recognized by T cells [114, 115]. Somatic mutations in cancer may give rise to mutated proteins that are degraded into peptides (neoepitopes) presented in the complex with major histocompatibility complex (MHC) molecules on the cell surface as neoantigens. There is a long standing interest in mutated antigens as discussed in a landmark review by Dr. Gilboa in 1999 [116]. Heroic efforts were made by multiple groups to assess reactivity against such antigens using DNA library screens. Although these studies illustrated proof of principle, it was not practically feasible to assess this class of antigen in a systemic manner. Only a minority of mutations are shared between patients; thus, the vast majority of mutated antigens are patient-specific. Therefore, the assessment of neoantigens needs to be based on the genome of individual tumors. The revolution in next-generation sequencing technology at affordable costs along with the progress in bioinformatics has now made it feasible to describe the full mutation load (i.e., the ‘genetic landscape’) of human tumors [117–120]. Specifically, a comparison of the genomic sequence of cancer tissue to that of non-transformed cells from the same patient can be used to reveal the full range of genomic alterations within a tumor, including nucleotide substitutions, structural rearrangements and copy number alterations [117].

Several preclinical and clinical reports underscore the importance of understanding the immunogenicity of neoantigens and their potential application in cancer immunotherapies. Two studies in mouse models provided the first evidence that cancer exome based approaches can be utilized to identify neoantigens recognized by CD8+ T cells [121, 122]. Moreover, a recent study showed that tumor specific mutant antigens are important targets of immune checkpoint blockade therapy [123]. Subsequently, it has likewise been demonstrated that similar approaches can be utilized in the clinical setting to identify immunogenic neoantigen specific CD8+ T cells in patients treated with tumor infiltrating lymphocyte (TIL) therapy and checkpoint targeting therapies [124, 125]. Two human studies reported that neoantigens were recognized by intratumoral CD4+ T cells in patients with epithelial cancer and melanoma [126, 127]. This accumulating evidence suggests that the immune response to mutant neoepitopes plays a dominant role in tumor rejection. Due to the uniqueness of neoantigens, research into tumor immunogenicity has shifted the interest from TAAs (differentiation antigen, cancer/testis antigen and overexpressed self-antigen) to patient-specific mutation antigens.

The studies in both mouse models and human material used exome sequencing, computer algorithm-guided epitope prediction and the *tandem minigene* library approach to identify MHC Class I- or II-binding neoepitopes that were processed and presented by APCs and recognized by neoantigen specific CD8+ and CD4+ T cells. A tumor harbors hundreds of putative neoepitopes per the analysis of the current TCGA database. It is imperative to differentiate and identify actual tumor protective neoepitopes from the putative neoepitopes defined in silico. There are two major factors that can be subject to variability when identifying tumor specific mutated antigens using these novel approaches. First, multiple computational tools to identify tumor specific mutations have been developed simultaneously. Different mutation calling tools such as EBcall, JointSNVMix, MuTect, SomaticSniper, Strelka and VarScan 2 have been developed to compare tumor samples with normal tissue samples at each variant locus in order to increase the accuracy of somatic single nucleotide variant (sSNV) calling. These tools used to identify mutations have a high degree of overlap [128, 129]. As a next step to identify neoepitopes, algorithms to predict binding affinity to patient specific HLA alleles can be used together with predictions on proteasomal processing. The accuracy of the prediction algorithms mostly depends upon calculating the score of binding to the MHC complex. Recent studies showed that combined use of multiple tools gave a better prediction [130–132]; however, more work is needed to accurately assess the immunoprotective properties of mutation-derived neoepitopes. Second, it has been demonstrated by unbiased screens that not all mutations result in neoantigens that are recognized by autologous T cells. Therefore, it would be valuable to have robust pipelines to filter whole exome data, especially for tumors with high mutation loads. Multiple groups have made significant efforts to establish such pipelines. The filtering steps that have been applied are based on the expression level of the mutations, e.g., RNA sequencing data, and the likelihood that a given mutated epitope will be processed by the proteasome and presented by patient specific MHC molecules [123, 125, 131, 133]. The two latter filtering steps can be assessed using algorithms that are already established to identify pathogen-derived epitopes. Currently, the data is still too sparse to know which of these filters is most relevant and how to accurately apply thresholds these filters to include immunogenic and exclude non-immunogenic neoepitopes. However, the most significant improvement in these predictions might be on the T cell side; the establishment of algorithms that can identify the subset of epitopes that are most likely to be recognized by TCR repertoire.

The development of robust in vitro T cell culture protocols, high-throughput combinatorial encoding of MHC multimer flow staining and high-throughput TCR gene capture allows us to assess the frequency, phenotype and polyfunctionality of the particular neoantigen specific T cell response [134–136]. These high-throughput technologies further reduce the large number of potential neoepitopes to a small number of real immunogenic neoepitopes. Therefore, these technologies will help us reevaluate the accuracy of computational tools as well as select candidate neoepitopes for vaccines and subsequently monitor the neoepitope specific T cell response during therapy. We will discuss the potential application of these high-throughput assays in the corresponding section. Overall, this approach, despite being in its early stages, has shown that the level of mutation load as a potential biomarker can correlate with clinical outcome to checkpoint blockade therapy in patients with advanced melanoma, colorectal cancer and NSCLC [62, 63, 104, 105, 137]. Patients with highly mutagenized tumors are most likely to respond to ipilimumab treatment. However, some melanoma patients with low mutation load have also experienced long-term clinical benefit. In addition, similar observations were reported in patients with NSCLC treated with anti-PD-1 antibody [104].

### Gene signature/pattern

Tumor immune biology is a complex interplay of many immunosuppressive and immune stimulatory components involve in connected pathways that define the inflammatory state of the TME. Single molecule perturbation in quantity and quality can induce a ripple effect under a given condition at a cellular and more importantly molecular level. Treatment induced coordinated changes or the natural course of the tumor can only be appreciated when all key components are examined simultaneously as a whole temporally and longitudinally. Evaluation of this complex interaction with respect to treatment outcomes in cancer have led to the identification of novel cell types that drive or contribute to the efficacy of or resistance to therapy and biomarkers that can predict clinical outcome or drive mechanisms of rejection [138–140]. A holistic evaluation of immune intervention in tumors can be achieved by a system biology approach using gene expression technology with high dimensional data analysis. As the technology become more affordable and reproducible with minimal input material, the type of sample that can be used for gene expression analysis ranges from fine needle aspirates, punch biopsies and needle sticks to laser capture microdissected (LCM) samples and archived samples with degraded RNAs. In addition, it makes the types of tissue usually available from clinical trials, such as formalin fixed paraffin embedded (FFPE) tumors, applicable. Combined with multiplexing IHC technologies, which allow for the comprehensive interrogation of multiple cell types as well as their location within the same tumor, gene expression is frequently used as a reasonable surrogate to identify the immune status of tumors [141–143].

However, reproducible and reliable expression data can only be achieved when the type of sample collection, timing of collection, sample processing and storage, standard laboratory procedures and platform selection are carefully planned. Several critical factors need to be considered when using gene expression as a tool for immune profiling. Two such factors are described below, including the platform technology and definition of gene signatures that signify the various immune cell subsets.

#### Sample quality and quantity

Because they most accurately represent directly ex vivo tissues, fresh samples should be the first choice for gene expression analysis. Because of advances in sample collection tools, the collection of fresh samples is now much less challenging. In order to monitor alterations in the peripheral blood immune profile, PAXgene RNA tubes (PreAnalytix GMbH, Hombrechtikon, Switzerland) or other similar products are ideal due to the direct preservation of the sample without the need for processing. For large volume blood collection, peripheral blood mononuclear cells (PBMC) should first be isolated followed by direct lysis into an RNA isolation reagent, such as QIAzol or Buffer RLT (QIAGEN, Venlo, Netherlands), depending on the isolation kit used. Alternatively, a cell pellet can be directly frozen in an RNA stabilization reagent. Ideally, tissue samples should be collected at bedside, immediately processed and submerged in an RNA stabilization reagent (e.g., RNA*later*, AMBION, Inc.). Time is a critical factor in sample collection, and this common step often introduces bias.

#### The technologies

For excisional biopsies, depending the tumor type and lesion, immune infiltrating cells are often quite diverse and comprise only a fraction of the total tumor mass. Consequently, RNA from immune infiltrating cells is generally poorly represented when enrichment methods such as LCM are not applied. Therefore, technologies that are capable of reliably detecting low abundance transcripts are the most suitable for the immune transcriptional profiling of human tumors.

RNA from FFPE tissues is often degraded and thus, poses a challenge for gene expression analysis. Recent advances in technology circumvent this challenge with the development of methods specialized for degraded RNA analysis. Of the various technologies available, the three most frequently used are digital PCR, single cell real-time PCR (using the Fluidigm BioMark or Nanostring nCounter analysis systems or Cytoseq technology), or whole transcriptome RNA sequencing. Each has its limitations and advantages [144–146]. The cDNA amplification step makes PCR the most sensitive technique listed above for measuring gene expression in immune cells. However, multiplex PCR is a tedious process and may consume significant amounts of RNA from precious tumor material. In contrast, digital PCR utilizes nanodroplet technology and makes multiplexing effortless. Alternatively, the Fluidigm BioMark system and Cytoseq technology utilize either a nanofluid approach or a combinatorial library of beads bearing cell- and molecular-barcoding capture probes and makes semi high-throughput real-time PCR possible and single cell profiling achievable. Although RNA or PCR amplification have been extensively used in molecular biology, amplification bias introduced during the multiple steps and enzymatic reactions can still affect data reproducibility. Given the lack of an amplification step, the Nanostring nCounter platform is the closest to representing the true copy number of mRNA and performs well for detection of RNA derived from FFPE tissues [147]. However, the lack of amplification may affect the sensitivity of the platform in detecting key immune cell transcripts particularly from cells that are poorly represented in tumors. The emerging front of high resolution whole transcriptome RNA sequencing is rapidly becoming the platform of choice for RNA profiling due to the affordable cost and in-depth resolution of data. It provides not only transcript copy number information, but also polymorphism information as well as transcript splicing variant information. The importance of splicing variants is becoming more and more appreciated and understood in terms of functional diversity and in relation to pathophysiology. Consequently, RNA sequencing of tumor samples is increasingly being used to identify neoantigens presented by MHC Class I molecules [148].

#### The definition of gene signatures

The power of gene expression platforms is in the ability to analyze genes in multiple cell types within a single experiment and to identify intrinsic immunosuppressive molecules and extrinsic inhibitory signatures, which may be predictive biomarkers and the targets for future immunotherapies. A simple strategy to evaluate multiple cell types is to incorporate lineage markers as representatives of the individual cell types. For example, CD20 and CD8 transcripts adequately represent B cell and CD8+ T cell densities, respectively. Cell types including tumor-associated macrophages, Th2 cells, and Treg among others are constantly changing both temporally and in response to changes in the microenvironment. These characteristics may be best studied using a gene signature approach. However, the ever changing microenvironment and cell dynamics makes generating reproducible gene signatures a moving target. Transcript analysis is a single snapshot of molecular activity at the point of sample collection and determined by multiple factors including the host’s genetic makeup, somatic genetic alterations, comorbidities, treatment procedures, protocol and time. Despite the evolving nature of the TME, common patterns of up or down regulated gene sets have been identified and validated in independent studies [140]. Gene analysis has evolved from pure gene signatures to identifying expression patterns based on pathway connections and molecules that are coordinated and associated with specific cellular phenotypes. Genes that are abundantly expressed in cells of interest tend to cluster together, thereby providing a surrogate readout for those cells. These gene signatures are often derived from the expression analysis of distinct individual cell types, representing the phylogeny of immune cells in terms of their differentiation [149]. One of the challenges of this approach in human tumors is the promiscuous expression of these genes in multiple cell types. For example, markers like perforin and eomesodermin (eomes) may represent both activated T cells as well as NK cells. Therefore, the preferred analysis method is to integrate data from multiple assays by correlating the results from different technologies, such as complementing gene expression analysis with flow cytometry staining and T and B cell receptor deep sequencing with multiplex IHC. Thus, this integrated approach yields a powerful method to accurately evaluate the immune profile of human tumors. The standardization of gene signatures that represent distinct immune cell types may be an important step in ensuring consistent interpretation of data from gene expression. Meta-analyses based on similar diseases and treatment regimens using public databases have been very fruitful in data validation and confirmation. Although a greater understanding of cancer biology and molecular immunology has been achieved, developing biomarkers to use in clinical practice would require further testing in large clinical studies and a broader database available for public access. To do so, extensive cross validation is necessary not only at a technological level, but also in interdisciplinary clinical trials.

### Epigenetic-differentiation based measurement of immune cell and other cell frequencies in blood and tissue using quantitative real-time PCR assisted cell counting

Epigenomics investigates key functional components that regulate gene expression in a cell, by providing information about patterns in which molecules such as methyl groups label DNA and histones. Epigenomic modifications provide a common set of instructions to achieve a cell type specific identity, despite sharing the same DNA sequence with all other cells in the body. Therefore, comprehensive epigenomic analyses can provide the missing link between genomic variation and cellular phenotype [150]. Epigenomic organization with cell type specificity is a major determinant of the cancer mutation landscape [151]. The National Institutes of Health (NIH) Roadmap Epigenomics Consortium established global maps of regulatory elements and defined regulatory modules of coordinated activity together with their likely activators and repressors. Thus explaining how cell-specific programs of gene expression are achieved and transcriptional and translational control is ensured. These data are a valuable resource for understanding the relationships between cells and tissues and interpreting the molecular basis of human disease [152].

One of these epigenetic modifications, the methylation status of either actively expressed or silenced genes, is the basis of a novel cell identification and quantification technology. Selective addition and removal of a methyl group to the 5′-carbon of the cytosine base occurs exclusively in the dinucleotide cytosine phosphate guanine (CpG). DNA methylation is a non-random event and often associated with inactive gene expression, if the target CpGs are located in the proximity of coding regions. In contrast, demethylation of CpG in regulatory elements is commonly accompanied by activation of gene expression. Recent discovery of cell type specific epigenetic CpG demethylation markers permits precise and robust quantification of immune cells from only small amounts of human blood or tissue samples.

These epigenetic biomarkers located on genomic DNA are stably associated with a cell type of interest. The cell quantification methodology is based on quantitative real-time PCR (qPCR), targeting differentially demethylated CpG marker regions in the genomic DNA revealed after a bisulfite conversion (BSC) step. During initial assay development, the cell type and subtype-specific epigenetic marker regions are identified through genome wide differential CpG demethylation analysis of highly purified reference cell populations of interest. The regions are selected based on specific DNA sequences with digitally differential BSC properties between different cell types. During BSC, unmethylated cytosines are converted to uracil, while methylated cytosines do not change. Therefore, respective CpG dinucleotides must be fully demethylated in the cell type of interest and methylated in all other cell types. Resulting determination of a cell type specific demethylation status in relevant loci is the basis for the development of segregating primer and probes. The readout technology is qPCR of the bisulfite converted DNA. Cell type specific qPCR assays are designed so that only the demethylated DNA is amplified. This facilitates subsequent fast quantification of various leukocyte and other cell populations in a given DNA containing sample by simple qPCR.

Representative examples are the complete demethylation of the Treg cell-specific demethylated region (TSDR) in Treg [153, 154], the demethylated region in the intergenic region of CD3D/CD3G in T cells [155] and the demethylation within the CCR6 locus in CCR6-positive cells [156]. Epiontis has identified, characterized and validated various epigenetic immune cell biomarkers, including those for Treg, Th17, Tfh, CD4+, CD8+ and CD3+ T cells, B cells, monocytes, NK cells and granulocytes. Results have an intra-assay coefficient of variation (CV) ≤15 % and inter-assay CV ≤20 %. Available assays cover the major leukocyte types and can be evaluated in regulated, clinical studies requiring a total of 2 ml or less whole blood for all markers combined. The amount of sample required for such studies is being lowered in the near future by a factor of 20 to about only 100 μl for all available assays together. Frozen tissue material requires 250 μg to 1 mg tissue to measure 12 markers, less for fewer markers. The exact material needed is less defined compared to blood because the number of cells and therefore DNA content in tissue by weight or volume varies significantly.

Due to the inherent stability of DNA and its markers, epigenetic assays have a distinctive advantage over assays that require intact or viable/functional cells in blood and tissue samples. This allows for a significantly broader range of acceptable sample conditions collected by clinical sites. By simply freezing and shipping the collected whole blood or tissue samples without any other additional steps, it allows for routine monitoring of patients and immune-monitoring during clinical trials, multicenter studies and retrospective studies as well as the comparison of results across different studies [157, 158]. A common application of this standardized, epigenetic-based, immune diagnostic technology is monitoring cell-mediated immunity during immune-modulatory clinical trials for cancer patients or inflammatory diseases. Because these tests can be applied on both blood and tissue, standardized measurements and a comparison of circulating and tissue-infiltrating immune cells can be obtained as an alternative to flow cytometry for peripheral blood samples and IHC for solid tissues. Future publication and extensive clinical studies are needed to validate the potential application of this novel technology for disease diagnosis and biomarker discovery for cancer immunotherapy. A Phase II immune modulation study has shown utility of Treg and CD3 cell monitoring in peripheral blood of patients in the SELECT trial [159].

### Protein microarray (seromics)

Proteomics, analogous to genomics, is the large-scale study of proteins, such as their structure, interactions and functions [160, 161]. Immunoproteomics is an extension of the proteomics field that studies immune related proteins and peptides. The release of tumor-derived proteins initiates an immune response that involves antigen-specific T and B lymphocyte targeting peptides binding to self-MHC molecules and generating specific antibodies to corresponding proteins. An autoantibody is an antibody that recognizes one or more proteins from an individual’s own cells. Autoantibodies in the peripheral blood are associated with autoimmune disorders, infectious diseases and cancer. An effective cancer immunotherapy would destroy tumor tissues, thereby releasing proteins and consequently priming T and B cells against additional tumor antigens that were not a part of the original therapy. This phenomenon is referred to as antigen spreading, or also known as epitope or determinant spreading [162, 163]. Therefore, the magnitude and spectrum of autoantibodies and their integration into the T cell response may be a feasible surrogate marker for measuring the adaptive immune response to cancer and a potential promising clinical biomarker.

Several immunoproteomics approaches, such as *Ser*ologic *P*roteome *A*nalysis (SERPA), *Ser*ological analysis of *re*combinant cDNA *ex*pression libraries (SEREX) and protein microarrays, have been investigated to identify TAAs and their cognate antibodies [164, 165]. SERPA is a classical immunoproteomic approach that provides a robust way of screening an antibody reactivity profile in sera from patients with various diseases. SEREX was used to discover tumor specific antigens that elicit a high titer immunoglobulin G (IgG) antibody in sera from patients with different types of cancer [166]. NY-ESO-1 was the first cancer testis antigen discovered by SEREX technology [167]. However, the application of SERPA and SEREX technologies is limited due to the assay specificity and the complexity of the assay preparation and procedure.

With the development of microarray techniques and thousands of purified proteins immobilized on a solid surface, protein microarrays have been employed to identify proteins, detect various protein binding properties, study protein posttranslational modifications and define potential biomarkers in a high-throughput manner. There are three major types of protein microarrays that are classified based on their technology and application: analytical, reverse-phase and functional protein microarrays [168]. The broader application of the first two types of protein microarrays is restricted by the specificity and availability of antibodies. Therefore, we will focus on the functional protein microarrays, especially commercial protein microarrays, to describe the advantages, current application and drawbacks of this technology in basic research and clinical studies.

Protein microarrays have several advantages compared with other techniques, including a reduction in sample volume used, high sensitivity and specificity and high-dimensional data generation. For example, Gnjatic et al. [169] reported that the sensitivity and specificity of a 329 full-length protein microarray had a 94 % concordance with a standard ELISA. Another commercial protein microarray, ProtoArray® (Life Technologies) offers a unique way to assay the serological response against thousands of proteins (~9,000) simultaneously. ProtoArray® does not have full coverage of the proteome, but serological analysis of this protein microarray represents a substantial portion of the human proteome (seromics). Integration of these types of high-dimensional data better representing the dynamic processes of the immunologic response associated with the development of the disease, related toxicity and clinical outcome to cancer immunotherapies. Specific autoantibodies have been shown to correlate with the status and tumor progression in patients with prostate, lung, ovarian and breast cancer [170–172]. In addition, humoral antigen spreading induced by Sipuleucel-T therapy was associated with improved overall survival [173]. Moreover, CTLA-4 blockade induced a broader antibody response in prostate cancer patients who responded to therapy compared with non-responders [56]. However, in another report, a prostate cancer patient who experienced a sustained complete response to CTLA-4 blockade mounted a strong humoral response against a small number of proteins, including one that is mutated in 5.5 % of prostate cancers [27]. Thus, further research using advanced methods will be necessary to fully understand the role of autoantibodies as a biomarker for cancer immunotherapy.

Similar to DNA microarrays, proper serum collection, sample storage and careful standard lab procedure for protein microarray analyses are required to avoid the inter- and intra-assay variation and improve data reproducibility. In addition, bioinformatics is critical for handling and processing the large datasets arising from these experiments. The analysis of protein microarray data involves six steps: data acquisition, pre-processing, visualization, differential analysis, result verification and computational feature annotation and network analysis [174]. Several software packages and computational tools have been developed for signal detection, data preprocessing, quality control and data normalization (see details in Table 1). A recent study proposed appropriate improvements on the default data analysis workflow [175]. Despite the ability of protein microarrays to generate substantial amounts of data for immune profiling in individual patients, the limitations of this technology, such as large-scale protein and antibody production, lack of label-free detection systems and relatively high cost, need to be overcome in the future. In addition, the target antibody/antigen identified from protein microarrays need to be validated by other technologies such as Western blot, ELISA, Luminex assays or mass spectrometry.

**Table 1:** Summary of novel technologies

Technology	Suggestions and potential biomarkers	Sample preparation	Bioinformatic tools	References and recommended reading
Whole exome sequencing for neoantigen discovery	• Mutation load for CTLA-4 and PD-1 blockade therapy • Neoantigen-specific T cell response	DNA from tumor and normal cells	EBcall, JointSNVMix, MuTect, SomaticSniper, Strelka, VarScan 2, BIMAS, RNAKPER SYFPEITHI, IDEB, NetMCHpan, TEPITOPEpan, PickPocket, Multipred2, MultiRTA	Van Buuren et al., 2014 [248]; Duan et al., 2014 [130]; Snyder et al., 2014 [62]; Snyder et al., 2015 [131]; Rizvi et al., 2015 [104]; Le et al., 2015 [105]; Van Allen et al., 2015 [63]
Gene signature and pattern	• MAGE-A3 gene signature • Chemokine expression in melanoma • Neoantigen signature	DNA and RNA from tumor, lymph node and PBMCs	BRB-ArrayTools, LIMMA, SAM, PAM, Partek, Genomic Suite, GSEA, Ingenuity IPA	Quackenbush et al., 2002 [231]; Simon et al., 2013 [249]; Simon et al., 2007 [250]; Subramanian et al., 2005; Smyth et al., 2005; Tusher et al., 2001 [251]; Tibshirani et al., 2002 [252]; Leek et al., 2010 [243]; Gaujoux et al., 2013 [245]; Ulloa-Montoya et al., 2013 [142]; Brown et al., 2014 [148]
Epigenetic-differentiation based immune cell quantification	• Immune cell lineage specific epigenetic modification • Leukocyte ratios in blood and tissue	Genomic DNA from fresh or frozen whole blood, PBMC, lymph node and fresh tissue or FFPE tissue and blood clots	HOMER package Motif Finder algorithm findMotifGenome.pl, MatInspector (Genomatix), Mendelian randomization	Wieczorek et al., 2009 [154]; Sehouli et al., 2011 [155]; Schildknecht et al., 2015 [253]; Steinfelder et al., 2011 [156]; Lavin et al., 2014; Gosselin et al., 2014; Liang et al., 2015
Protein microarray (seromics)	• TAA antibody response • Broad antibody signature • New antigen discovery	Fresh or frozen serum and plasma	Prospector, LIMMA package, PAA package, Spotfire package	Gnjatic et al., 2009 [254]; Kwek et al., 2012 [56]; Turewicz et al., 2013 [175]; Graff et al., 2014 [27]
Flow Cytometry and Mass Cytometry	• Use best flow practices and recommended flow panels • Multimers for T cell epitope screening • TAA-specific T cell response for CTLA-4 blockade therapy • CD4+ICOS+ T cells for CTLA-4 blockade therapy • Baseline MDSC for CTLA-4 blockade therapy	Whole blood; Fresh or frozen PBMCs and TILs; Fresh or frozen cells from ascites or pleural effusion	Computational algorithm-driven analysis for MDSC, Cytobank, FlowJo, SPADE, PhenoGraph, PCA, viSNE, Citrus, ACCENSE, Isomap, 3D visualization	Maecker et al., 2010 [176]; Maecker et al., 2012 [177]; Streitz et al., 2013 [178]; Kvistborg et al., 2012 [255]; Chang et al., 2014 [190]; Yuan et al., 2011 [57]; Carthon et al., 2010 [50]; Kitano et al., 2014 [72]; Levine et al.,2015 [189]
T and B cell receptor deep sequencing	• CD3 T cell count • T Cell clonotype stability for CTLA-4 blockade therapy • Baseline T cell clonality in tumor in PD-1 blockade therapy	DNA from FFPE; Frozen cells from tumor, lymph node or PBMCs; Fresh or frozen cells from ascites or pleural effusion	Shannon Entropy, Morisita’s distance, Estimated TCR gene rearrangements per diploid genomes, Clonality, ImmuneID, Adaptive ImmunoSeq software	Cha et al., 2014 [205]; Tumeh et al., 2014 [103]; Howie et al., 2015 [202]
Multicolor IHC staining	• CD3 Immune score • CD8/FOXP3 ratio for tumor necrosis • PD-L1 expression on tumor in PD-1 blockade therapy	FFPE tissue; Fresh or frozen tissue	TissueGnostic system, PerkinElmer system	Galon et al., 2006 [10]; Hodi et al., 2008 [54]; Taube et al., 2014 [110]

*Abbreviations*: *PBMC* peripheral blood mononuclear cells, *TAA* tumor associated antigen, *MDSC* myeloid derived suppressor cells, *TILs* tumor infiltrating lymphocytes, *IHC* immunohistochemical staining, *TCR* T cell receptor, *FFPE* formalin-fixed, paraffin-embedded, *PD-1* programmed cell death-1, *PD-L1* programmed cell death ligand −1

### Flow cytometry and mass cytometry

Since its inception, flow cytometry has been a powerful technique for the field of immunology because of its unique ability to analyze large numbers of single cells with multiple parallel probes. This allows for the identification as well as the deep phenotypic and functional analysis of rare subpopulations of cells. The lack of standardization in flow cytometry has traditionally hindered its application in multicenter clinical trials. However, there have been recent efforts to recommend best practices for such multicenter studies [176]. Standardized panels have also been published for PBMC or whole blood immunophenotyping [177, 178], and for leukemia and lymphoma diagnoses [179]. Moreover, the number of available fluorochromes for flow cytometry has steadily increased. Recent years have seen the advent of so-called “Brilliant” dyes, or π-conjugated polymers, which are bright (due to cooperative energy transfer) and have tunable emission wavelengths [180]. These dyes have fostered a quantum leap in the ability to do multicolor flow cytometry, making 12–15 colors not only feasible, but routine.

During the same timeframe, mass cytometry (or CyTOF, for Cytometry by Time of Flight) has emerged as a competitive platform for high-dimensional single-cell analysis [181, 182]. This technology uses probes that are labeled with heavy metal ions via covalently coupled chelation polymers, rather than fluorescent probes. The subsequent readout by mass spectrometry allows for the simultaneous detection of many more unique probes, with little or no spillover between detector channels [183]. The current state of the art is about 40 parameters per cell, with both phenotypic and functional assays developed [184–189].

The main drawbacks of mass cytometry include slow collection speed (about 300 events/s), low recovery of cells in the instrument (typically 30 %), and expense. To some extent, these drawbacks are mitigated by the ability to stain a single tube rather than create a panel of several tubes for conventional flow cytometry, which requires more cells, time, and reagents. While the sensitivity depends on the choice of label, instrument setup, and other factors, there are limitations to the sensitivity of CyTOF, as no channel can provide an equivalent resolution sensitivity to the brightest conventional fluorophores. This, too, may be mitigated by the ability to resolve populations in many more dimensions, but it remains a limitation at the single-marker level.

One application of highly multiparameter cytometry, especially mass cytometry, is for the broad analysis of immune competence in cancer patients. Given the present rise in use of immunotherapy and its reliance on the immune system to provide a response, it is surprising that comprehensive and standardized measures of immune competence are not more frequently performed. By simultaneously probing the phenotypes and functions of multiple immune cell subsets in a single PBMC sample, mass cytometry can provide a “fingerprint” of immune responsiveness, which may eventually yield correlates of responsiveness to therapy [190]. Eventually, this concept could be expanded from measurement of mitogen-stimulated responses to parallel measurement of specific T cell responses to multiple tumor antigens. It could also be used to measure immune cell phenotypes and functions in tumor biopsies, which are likely to be more informative than PBMC for predicting patient responsiveness.

The delineation of T cells into distinct functional populations defines the quality of immune response which is crucial to disease outcome [191]. Multiple functional parameters including cytokine, chemokine and degranulation in response to antigen specific stimulation can be simultaneously detected by flow cytometry. Polyfunctional T cell responses have been demonstrated to correlate with clinical response in patients with infectious disease and in cancer patients treated with immunotherapy [192–194]. Another attractive application of multiparameter cytometry is the use of peptide-MHC multimers to identify T cells of a given specificity or to screen for multiple specificities. To date, combinatorial multimer analyses have been performed using both flow and mass cytometry and allowed screening for 145 and 109 T-cell specificities, respectively [135, 195]. The strategy was key to demonstrate that treatment with ipilimimab enhanced the priming of new T-cell responses rather than boosting preexisting responses [61]. Similarly, the usage of MHC multimers is also likely to become a powerful tool for epitope discovery, allowing high-throughput identification, enumeration and profiling of neoantigen-specific T cells.

### T and B cell receptor deep sequencing

Advances in high-throughput sequencing have enabled the development of a powerful new technology for probing the adaptive immune system called immunosequencing [196–199]. Millions of B or T cell receptor (BCR or TCR) sequences can be read in parallel from a single sample. Each B or T cell clone has a unique (or nearly unique) adaptive immune receptor generated through a highly regulated process of somatic DNA rearrangement. When the BCR or TCR of a clone binds its target antigen as part of an immune response, the clone divides rapidly, called clonal expansion. Unlike sequencing human (or human cancer) genomes, immunosequencing must be quantitatively accurate, because the adaptive immune system works by the principle of clonal expansion.

Despite the new challenge of quantitatively sequencing a highly variable, complex locus, the field has developed quite rapidly. A few techniques have been developed to accomplish the quantitative sequencing of the loci and standardize the methods, even between laboratories [200–202]. In the next year or two, the use of immunosequencing for diagnosis and monitoring of lymphoid malignancies is expected to achieve FDA approval and CE mark [203, 204].

There are many potential applications of immunosequencing in immunotherapy. Because each clone has a nearly unique sequence, T cell clones can be tracked over time, between tissues and between phenotypic subsets. This technology is helping researchers understand the mode of action and differences between therapeutic agents. For example, when comparing melanoma tumor samples before and after anti-PD-1 therapy, the primary T cell clonal expansions are from clones present prior to therapy [103]. This suggests that anti-PD-1 therapy primarily enhances and/or unblocks a pre-existing immune response in the tumor as opposed to inducing a new response. Although, this was suggested as a mode of action, immunosequencing was required for solid evidence.

A set of potential immunosequencing biomarkers in immunotherapy are presently being explored. These include predictors of response to therapy as well as monitoring of pharmacodynamics changes, drug efficacy and side effects. The predictive biomarkers can be dividing into two groups. The first group is measuring immunosequencing of tumor infiltrating lymphocytes (TIL). A recent study has shown that both the number of TIL and degree of specific clonal expansions (a telltale sign of an adaptive immune response) in pre-treatment melanoma samples is predictive of response to anti-PD-1 therapy [103]. There is ongoing work to confirm the findings in larger cohorts and other tumor types. Additionally, similar biomarkers are being evaluated for different immunotherapeutic agents [205]. Importantly, such biomarkers have the potential to help guide combination therapies or dose regimens [206]. If a particular TIL signature is needed for efficacy of anti-PD-1 therapy, then other therapies that can generate the TIL signature would be likely combination candidates. The advantage of immunosequencing is that the activation of infiltrating killer T cells are thought to be the mode of action for checkpoint inhibitor therapy, so TILs are a causative biomarker, not just a correlative biomarker. Many of the practical issues associated with tumor samples have been (or are presently being) addressed. Tumor heterogeneity (sampling) does not appear to be a significant issue relating to TIL [207, 208]. With the proper controls, FFPE can be readily utilized in this assay.

Less direct, although potentially practical and broadly applicable, is the second group of biomarkers, which are blood based. There is increasing evidence that the distribution of T cell clones in the blood is a correlate of immune competence. If a patient’s immune system is not functioning properly, an immunotherapy is unlikely to be successful. In addition, unregulated responses, such as autoimmune reactions are more likely [209].

For other types of immunotherapy, such as adoptive T cell transfer or chimeric antigen receptor T cell (CAR-T) therapy, immunosequencing is used to monitor the therapy itself by tracking the injected T cells. Moreover, when the target is a lymphoid malignancy, immunosequencing is commonly utilized to monitor minimal residual disease post treatment [210]. The breadth of potential immunosequencing biomarkers in immunotherapy is very large. Immunosequencing is a molecular, reproducible tool for evaluating the adaptive immune system in humans, which has opened many avenues of biomarker research.

### Multicolor IHC staining

The detection of structural and functional proteins along with their spatial localization within cells or extracellular compartments in tissue samples is achieved by immunolabeling with specific antibodies. The antibody binding is then detected with the application of either an enzymatic reaction that induces chromogen precipitation at the site of antibody-antigen binding (immunoenzyme method) or by using fluorescent dyes (fluorochromes, fluorescent quantum dot nanocrystals), conjugated either to primary or secondary antibodies (direct or indirect immunofluorescence, respectively).

For routine histopathological diagnosis, the immunoenzyme methods offer the advantage of permanent staining by using panels of antibodies (1 reaction/section) specific for a tumor subtype, while optimal morphology is maintained. Rarely, double staining protocols are applied to evaluate the ratio of two different cell types or to evaluate pairs of antigens localized in different intracellular compartments, e.g., the cytoplasm or nucleus. However, in order to maximize the information that can be acquired from the intact tumor anatomy and delineate any spatial and temporal (pre- versus post-treatment) heterogeneity that influence tumor biology and could be used as a biomarker, multiplexed staining approaches and imaging systems are required. Practically, multiplex approaches constitute repetitions of individual immunolabeling methods, either applied in one step or in sequential rounds [211]. These methodologies suffer from inherent problems that have to be overcome, such as cross-reactions between individual stains and limitations regarding the color (chromogens or fluorochromes) combinations. Aside from using primary antibodies from different species, cross-reactions are prevented in immunoenzyme methods by heat-induced removal of primary antibodie(s) from the previous staining round, whereas the heat-stable chromogen remains and tags the antigen target. For these chromogenic methods, 3–4 colors appear to be the multiplex limit.

Alternatively, for multiplexed immunofluorescence one can perform the tyramide signal amplification technique, in which fluorophore-labeled tyramide, upon activation by horseradish peroxidase, covalently binds to tyrosine residues of proteins adjacent to HRP-conjugated antibodies. Following the heat-induced removal of antibodies from the previous round, a new individual stain can be applied, with a minimal risk of cross-reaction. This technique is important for fluorescent multiplexing. After the completion of the multiplexed staining procedure, up to 5 fluorescent dyes can be evaluated using common fluorescent microscopes with standard optical filter cubes or up to 8 fluorescent dyes can be evaluated using microscopes equipped with a multispectral camera [212].

Other multiplexed methods that could circumvent the limitations of overlapping emission spectral of fluorescent microscopy are based on successive cycles of antibody tagging, imaging and removal/bleaching of the fluorophore(s) [213, 214]. With dedicated software, these hyperplexed approaches have been used to characterize multiple analytes (>100) in the same section. Interestingly, recently described multiplexed methodologies have been performed with antibodies labeled with metal isotopes and analyzed by mass cytometry-based approaches [215, 216].

Overall, these in situ multiplexed methods add greater depth to our understanding of tumor pathogenesis. Furthermore, when applying these methods to immunity-related analytes in the TME by combining phenotypic and functional markers with specific spatial point-pattern analyses, these methods will increase our knowledge of underlying mechanisms that could be used to optimize the efficacy of immunotherapy protocols.

### Three-dimensional (3D) cell culture models

Traditionally, cell-based assays to explore cell biology and drug efficacy have been aimed at growing cells on two-dimensional (2D) plastic surfaces or in single cell suspensions [217]. However, the cellular biology is profoundly influenced by the microenvironment. Thus, cell based assays are needed that reflect the effects of factors such as the extracellular matrix, cell-cell contacts, and cell-matrix interactions [218–221]. Not only the cell morphology but also the drug sensitivity of cancer cells in 2D systems have been shown to be different compared with 3D cell cultures [6, 7]. Cells cultivated on plastic surfaces usually exhibit an increased sensitivity to cytotoxic drugs, while compounds targeting cell-cell adhesions, cell maturation, epithelial-mesenchymal transition and stemness often show a decreased efficacy in 3D cell culture systems. Thus, 3D cell culture models reflect in vivo tumor growth more reliably and may provide better readouts for drug testing [222–224].

The hanging drop technique is a well-established cell culture method to form spherical microtissues from immortalized and primary cell lines [225–227]. In contrast to most liquid overlay technologies, the hanging drop method allows the precise control over the initial cell composition in each microtissue [228, 229]. In addition, to generate multicellular co-culture microtissues, neither additional supplements nor artificial scaffolds mimicking extracellular matrix components (e.g., collagen matrigel) are required. A 3D hanging drop system has been established for cancer cell lines that are cultivated either alone or together with a lung fibroblast cell line to investigate tumor stroma interaction [230]. In this model, IHC, flow cytometry, epifluorescence, confocal and scanning electron microscopy can be performed to assess alterations not only in protein expression and viability but also in microtissue aggregation. The addition of endothelial cells to these co-cultures of cancer cells and fibroblasts can further mimic an in vivo environment.

Upregulation of mesenchymal markers and downregulation of adhesion molecules can be observed in multicellular microtissues compared with 2D monocultures. Moreover, a difference in Ki67 expression indicates different states of high metabolic activity. In addition, changes in the morphology of tumor cells can be achieved, particularly in microtissues cultured with endothelial cells. Immune cells from freshly isolated PBMCs can be added to microtissues in the 3D hanging drop technology. After short or long term co-incubation, the microtissues are “harvested” and immune cell penetration into the spheroids is analyzed. In this model, cytokine stimulation (e.g., IL-2) of immune cells leads to a significantly increased ability of immune cell migration into the microtissue and induction of cellular cytotoxicity.

The 3D cell model represents a promising approach for defining and analyzing immune-based biomarkers, as it provides an ex vivo approach to measure immune cell function in an organotypic culture model. In addition, this method is uniquely advantageous in that primary tumor cells and autologous immune cells from individual patients can be used in this model. Although there is a certain limitation to this system as it does not fully model a whole organism, the 3D culture system could be combined with studies in mouse models to overcome this disadvantage.

### Bioinformatic tools and data analysis for high-throughput data

As high-throughput technologies become more standardized and widely available, bioinformatics tools to analyze, interpret, and visualize data will be in high demand. Several tools and software packages have been developed to mine data and extract useful information from large datasets. A list of commonly used bioinformatics tools for different high-throughput technologies is summarized in Table 1.

There are many key steps involved in the analysis of high-throughput data. For example, data normalization is a critical step to reduce the intra- and inter-array variability caused by systematic artifacts without losing useful biological information. Different normalization approaches have been extensively studied for data generated from various high-throughput platforms, e.g., gene expression arrays, miRNA arrays, protein microarrays, and RNA-sequencing experiments. For each high-throughput platform, comparisons have been made between the different normalization methods, such as global normalization, Lowess normalization, quantile normalization or conditional quantile normalization, variance stabilizing normalization, Z-Score normalization and robust linear model normalization [231–241]. For epigenomic data such as the epigenetic regulation of immune cells, data preprocessing and normalization includes inverse normal transformation or Z-score normalization. In general, it is recommended that the assumptions that underlie each normalization method are carefully considered and evaluated in order to determine which method is most appropriate for the experimental setting. In addition, more than one normalization approach should potentially be considered in order to determine the sensitivity of the results to the normalization method used.

Batch effects are commonly found in high-throughput technologies due to the influence of laboratory conditions, reagent lots and personnel differences. When batch effects correlate with an outcome of interest, they can be very difficult to detect and remove, which can easily lead to false conclusions. Several approaches have been proposed to correct for batch effects, and many, such as ComBat or SVA, have been shown to be effective [242, 243]. In order to manage a potential batch effect, the first step is to identify and quantify it using principle components analysis or visualization techniques, such as hierarchical clustering or multidimentional scaling. Once strong batch effects are identified, the subsequent analyses must be adjusted to account for these effects.

Because the tissue samples used for mRNA extraction are often a heterogeneous mix of cell types, identifying biologically relevant, differentially expressed genes in order to develop and validate predictive models is very difficult. Thus, several statistical approaches have been proposed to deconvolute gene expression profiles obtained from heterogeneous tissue samples into cell-type-specific subprofiles [244–246]. CellMix is one such package designed for the R statistical suite that incorporates state of the art deconvolution methods into an intuitive and extendible framework to explore, assess and disentangle gene expression data from heterogeneous samples [245].

### The challenge for future biomarker discovery

We have discussed the challenges in individual high-throughput assays and in the subsequent bioinformatic analysis of high-throughput data above. Therefore, we would also like to highlight a few general challenges for future biomarker study in the following section.

#### Specimen type

Peripheral blood is commonly used for biomarker studies because of its easy accessibility. Several emerging, potential biomarkers discussed in this paper were identified in peripheral blood using validated assays. One of the advantages of using peripheral blood is that pharmcodynamic changes can be measured at multiple time points before and after mono or combination therapy. However, there is still a major question as to whether what we learn from PBMCs is relevant to solid tissues. For example, T cell clonal expansions are local due to the enriched selective lymphocyte population and immunological response to immunotherapies. Thus, expanded T cells that can be detected in the tumor may not be present in the peripheral blood.

In general, fresh tumor tissue is of higher quality compared with frozen tissue. However, getting access to fresh tissue, especially prior to treatment, is challenging. A pre-treatment tissue biopsy may be a requirement for enrollment in a clinical trial, but this is not always done. In addition, tissue obtained in second-line therapies may not be the most informative due to the impact of multiple chemotherapies. The amount of tissue required for TCR analysis (a few micrograms or approximately 20 μm of slices) is relative low. However, the number of TIL in a tissue block is highly variable, which can make it difficult to obtain statistically robust results.

#### Standardizing procedures and assay validation

The application of a potential biomarker in a clinical setting requires several layers of validation as described above, including a standardized specimen banking procedure as well as assay validation and confirmation in a randomized, large-scale clinical study. The importance of the immunoprofile in colon cancer and other malignancies is evident. Thus, well-validated, consistent protocols for the collection and cryopreservation of tumor material are necessary to avoid introducing potential variation during sample collection and storage.

Preserving an adequate amount of fresh tissue is the key to obtaining a sufficient amount of immune cells. In the past, a major limitation was the size of the fresh tumor sample and the number of cells that could be extracted (with or without enzymatic digestion) from it. Therefore, it was essential to calculate the minimum number of viable cells needed in order to receive reliable results for each type of assay or biomarker being studied. Recent advances in novel high-throughput technologies have provided a solution to this problem. These new technologies can decrease the amount of tissue needed while still providing reliable results and a vast amount of data. One slide can provide plenty of information with the use of new programming systems that can quantify and present different types of cells based on markers in standard flow cytometry plots [247]. Conventional flow cytometry assays require approximately 5 × 10^6^ cells. However, microfluidic devices may eventually be able to handle very small cell numbers, e.g., 500 cells, without significant loss. These novel flow-based technologies can assess the phenotype and multifunctionality of tumor-specific T cells in samples containing few cells and correlate these findings with clinical outcome. However, it is challenging to standardize and validate all of the developing high-throughput technologies, especially across multicenter clinical trials. Thus, a cooperative effort will be necessary for future biomarker validation.

#### Funding, resources, and collaboration

In order to advance future personalized cancer immunotherapies, there is a trend toward high quality, mechanism-based translational research using well-validated, high-throughput immune assessments. However, this type of research requires specialized equipment, well-trained staff, professional statistical analysis and data sharing that all come at a high cost. Therefore, a continuous effort is needed to generate public awareness about the importance of supporting this research through increased funding from governmental and nonprofit organizations. In the last several years, results from prior translational biomarker studies have provided valuable guidance for late-phase clinical trials. In addition, the pharmaceutical and biotech industry has recognized the importance and value of biomarker-based research, emphasizing the need for proper sample collection procedures, well-validated high-throughput assays and experienced scientists for accurate data interpretation. Productive translational collaborations between academia and industry have advanced biomarker development and the clinical development of cancer immunotherapies. One particular future area of interest would be a cross-sector collaboration to develop a cooperative proposal in order to standardize biomarker research in clinical trials.

## Conclusions and recomendations

As a result of over a century’s efforts to understand the role of the immune system in controlling cancer, immunomodulation by checkpoint inhibitors (targeting both CTLA-4 and the PD-1/PD-L1 axis) induced a durable tumor response in a wide range of malignancies. In addition to the FDA approvals of single immune checkpoint blockades for cancer immunotherapy, the FDA has granted accelerated approval to the combination of nivolumab and ipilimumab to treat advanced melanoma, the first approval of any immunotherapy combination to treat cancer. These therapies are revolutionizing therapeutic concepts and changing the standard of care for cancer treatment. Immunotherapy is now widely accepted as a key component of the therapeutic strategies to control and potentially cure cancer. Moreover, immunotherapy has the potential to cure or convert cancer from a fatal disease into a non-life threatening or chronic disease. The concept of a “clinical cure” is emerging as a description of long-term tumor control. The broad potential for a clinical cure is now being extensively explored by both mono and combination cancer immunotherapy.

The complexity and heterogeneity of the interaction between the immune system and tumor cells, particularly in the TME, underlies the immune status (i.e., immunologically responsive or immunologically ignorant) of each individual tumor for every patient. Biomarker-based research is an essential approach to understand both intrinsic and extrinsic tumor escape mechanisms. Recent advances in technologies have provided tools that will facilitate an in-depth understanding of this interaction and will help guide the development of future personalized cancer immunotherapies. Whole exome sequencing allows mutation load to be assessed in each individual tumor; prediction algorithms and the *tandem minigene* library enables the identification of both class I and II neoepitopes, respectively. Novel gene expression technologies can be used to accurately identify the immune status of tumors from properly collected and processed specimens. B and T cell receptor deep sequencing provides the full spectrum of the B and T cell receptor repertoire and can be used to potentially identify immunosequencing biomarkers in both peripheral blood and tumor tissue. Classic immune monitoring assays, such as ELISpot, tetramer and intracellular cytokine flow cytometry staining, are still useful to assess tumor antigen specific T cell response, especially neoantigen specific T cell responses after neoantigen vaccination or immune checkpoint blockade therapy. Multi-color IHC staining provides spatial localization and distribution of phenotypic and functional biomarkers within the TME. Gene microarray, deep sequencing technologies, flow cytometry staining and IHC staining can typically be performed by core facilities in academic centers or biotech companies. However, novel technologies that are at earlier stages of development, such as mass cytometry, are not widely accessible yet.

High-dimensional data generated from these novel innovative technologies can be generally classified into three types: function, phenotype and signature/pattern. It is of importance to evaluate the function of cytolytic T cells, especially in the TME. Phenotype data provides the frequency and status of different immune cells and their potential impact on cytolytic T cells. Signature/pattern results will help elucidate the potential mechanisms of action and guide future biomarker research. Currently, the high cost of these technologies limits the number of assays that can be performed for each study. However, in order to obtain multidimensional data to get a complete picture of the immune status of the TME, it may be best to perform a combination of assays in order to obtain these three types of data as described above.

The potential biomarkers and new technologies discussed here from exploratory studies need to be validated in future clinical studies. Overall, the ideal biomarker should be convenient to use in a clinical setting and provide an accurate prediction of a patient’s clinical response. In addition, new knowledge obtained from ongoing studies and emerging technologies will refine our strategy for the practical clinical application of biomarker research.

## Abbreviations

SITC: society for immunotherapy of cancer
WG2: working group 2
TME: tumor microenvironment
PD-1: programmed cell death protein 1
PD-L1: programmed cell death ligand 1
CTLA-4: cytotoxic lymphocyte-associated antigen 4
Tim-3: T cell immunoglobulin mucin-3
LAG-3: lymphocyte-activation gene 3
IDO: indoleamine 2,3-dioxygenase
Treg: regulatory T cells
MDSC: myeloid derived suppressor cells
NSCLC: non-small cell lung cancer
TCR: T cell receptor
BCR: B cell receptor
FDA: food and drug administration
LDH: lactate dehydrogenase
VEGF: vascular endothelial growth factor
HLA: human leukocyte antigen
ICOS: inducible co-stimulator
TAA: tumor associated antigen
ALC: absolute lymphocyte count
APCs: antigen presenting cells
nTreg: naïve regulatory T cells
tTreg: thymus-derived regulatory T cells
iTreg: inducible regulatory T cells
pTreg: peripheral regularoty T cells
LAP: Latency-associated peptide
GARP: glycoprotein A repetitions predominant
CFSE: carboxyfluoresceinsuccinimidyl aster
KLF2: Kruppel-like factor 2
TGF-β: transforming growth factor-beta
IL-10: interleukin 10
FOXP3: forkhead box protein 3
ITAC: inducible T-cell alpha chemoattractant
IHC: immunohistochemistry
TIL: tumor infiltrating lymphocyte
LCM: laser capture microdissected
FFPE: formalin fixed paraffin embedded
PBMC: peripheral blood mononuclear cells
NIH: National Institutes of Health
CpG: cytosine phosphate guanine
BSC: bisulfite conversion
TSDR: regulatory T cell-specific demethylated region
CV: coefficient of variation
MHC: major histocompatibility complex
SERPA: serologic proteome analysis
SEREX: serologic analysis of recombinant cDNA expression libraries
CyTOF: cytometry by time of flight
CAR-T: chimeric antigen receptor T cell
3D: three-dimensional
